# Polymer clip granuloma mimicking lymph node recurrence: a case report

**DOI:** 10.1186/s40792-024-01881-2

**Published:** 2024-04-02

**Authors:** Shiori Kimura, Masaki Honda, Yohei Sanmoto

**Affiliations:** Department of Surgery, Takeda General Hospital, 3-27 Yamaga-machi, Aizuwakamatsu-shi, Fukushima, 965-8585 Japan

**Keywords:** Foreign body granuloma, Polymer clip, Lymph node recurrence, Postoperative, Surgical material

## Abstract

**Background:**

Foreign body granulomas are postoperative inflammatory reactions to surgical materials within the body. Traditionally, sutures have been the leading cause of foreign body granulomas in the abdomen, commonly referred to as “suture granuloma”. However, the use of polymer clips in modern surgeries has introduced new challenges, and reports of intra-abdominal foreign body granulomas caused by surgical clips are limited. Herein, we present a case of an intra-abdominal foreign body granuloma formed due to polymer clips.

**Case presentation:**

A 45-year-old woman who underwent laparoscopic resection for sigmoid colon adenocarcinoma subsequently developed a suspected lymph node recurrence during follow-up. Imaging showed an enlarging mass adjacent to the inferior mesenteric artery with increased fluorodeoxyglucose uptake. Laparoscopic surgical resection revealed the formation of a foreign body granuloma in response to the polymer clips.

**Conclusions:**

This case suggests that nonabsorbable polymer clips can induce granulomatous reactions postoperatively, and the appearance of lymph node recurrence may be foreign body granulomas.

## Background

Foreign body granulomas are inflammatory reactions that may occur postoperatively when surgical materials are left inside the body. Conventionally, silk sutures are the primary cause of intra-abdominal foreign body granulomas [1–7]. They can be incidentally discovered on computed tomography (CT) images in patients who have undergone cancer surgery, occasionally leading to a misdiagnosis of cancer recurrence [1–6]. Such misinterpretations can result in unnecessary invasive treatments, such as systemic chemotherapy [8] or high-risk extended operations, including pancreatoduodenectomy [9].

Currently, polymer clips are commonly used for vessel ligation, particularly in laparoscopic and robotic surgeries. Despite their increased use, reports of intra-abdominal foreign body granulomas caused by surgical clips are limited. Combined with a review of the previous literature, we present a case in which suspected lymph node recurrence after sigmoid colon adenocarcinoma resection turned out to be a foreign body granuloma formed due to polymer clips.

## Case presentation

**Fig. 1:**
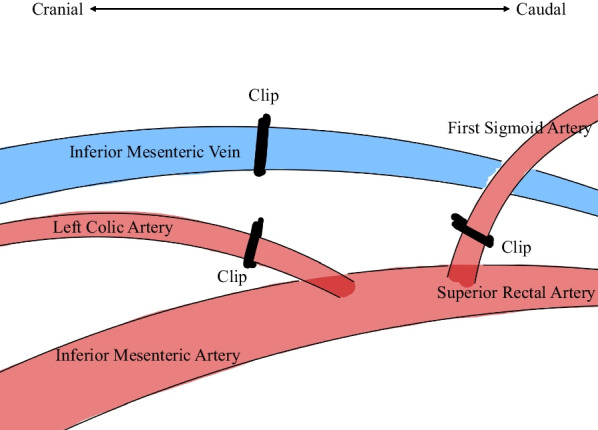
Surgical anatomy of the vessels during initial surgery. The left colic artery, first sigmoid artery, and inferior mesenteric vein are ligated using polymer clips

A 45-year-old woman with no significant medical history or allergies was admitted to our hospital with severe microcytic anemia. Subsequent colonoscopy revealed a type 2 tumor in the sigmoid–descending junction, which was pathologically diagnosed as a papillary to well-differentiated tubular adenocarcinoma. Combined with contrast-enhanced CT findings, a preoperative diagnosis of sigmoid colon cancer without metastasis (cT2N0M0, cStage I according to the Union for International Cancer Control TNM classification) was established, and laparoscopic left hemicolectomy with D2 lymph node dissection was performed. Surgical findings indicated no signs of metastatic disease, liver nodules, or peritoneal implants. First, the left colon was mobilized using a medial approach, scoring the mesentery between Gerota’s and Toldt’s fasciae. The inferior mesenteric artery (IMA) and superior rectal artery (SRA) were skeletonized by dissecting the lymphatic adipose tissue along them. The left colic artery (LCA) and the first sigmoid artery (S1) were then ligated with polymer Hem-O-Lok® clips (Teleflex), while the SRA was preserved (Fig. 1). The inferior mesenteric vein (IMV) was ligated in the same manner at the level of the LCA. The colon was fully mobilized for transection at the splenic flexure and sigmoid colon. After the resection, extracorporeal functional end-to-end anastomosis was performed. Later, the pathological sample was reported to be a carcinoma with an adenoma component (pT1N0M0, pStage I). Of the total 20 #241 and #242 lymph nodes examined, none exhibited malignancy. The patient was discharged on postoperative day 7, and her tumor marker test at 3 months postoperatively was insignificant, with a carcinoembryonic antigen (CEA) level of 4.2 ng/mL. Subsequent monitoring every 3 months, including CT and lower gastrointestinal endoscopy, revealed no evidence of recurrence, except for a slightly elevated CEA of 6.7 ng/mL, likely due to smoking resumption.

**Fig. 2:**
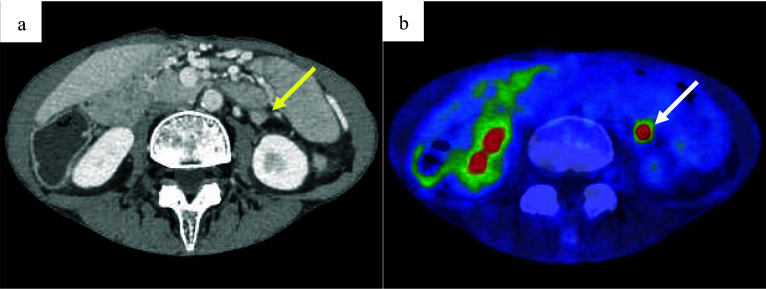
Follow-up contrast-enhanced computed tomography (CT) and positron emission tomography (PET)/CT 2 years after the initial surgery. **a** Contrast-enhanced CT shows a 10 mm mass (arrow) adjacent to the inferior mesenteric artery. **b** PET/CT scan reveals a mass (arrow) at the same location with elevated fluorodeoxyglucose uptake, indicated by a maximum standardized uptake value of 10.6

**Fig. 3:**
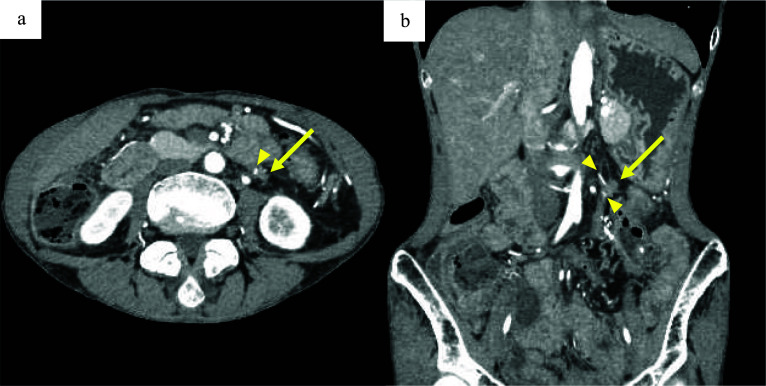
Follow-up preoperative contrast-enhanced computed tomography (CT). Preoperative contrast-enhanced CT shows an enlarged mass (arrow) with embedded clips (arrowheads). **a** Axial view of the 15 mm mass, 5 mm larger than that seen in the initial follow-up CT, performed 3 months before. **b** Coronal view of the mass adjacent to the inferior mesenteric artery

**Fig. 4:**
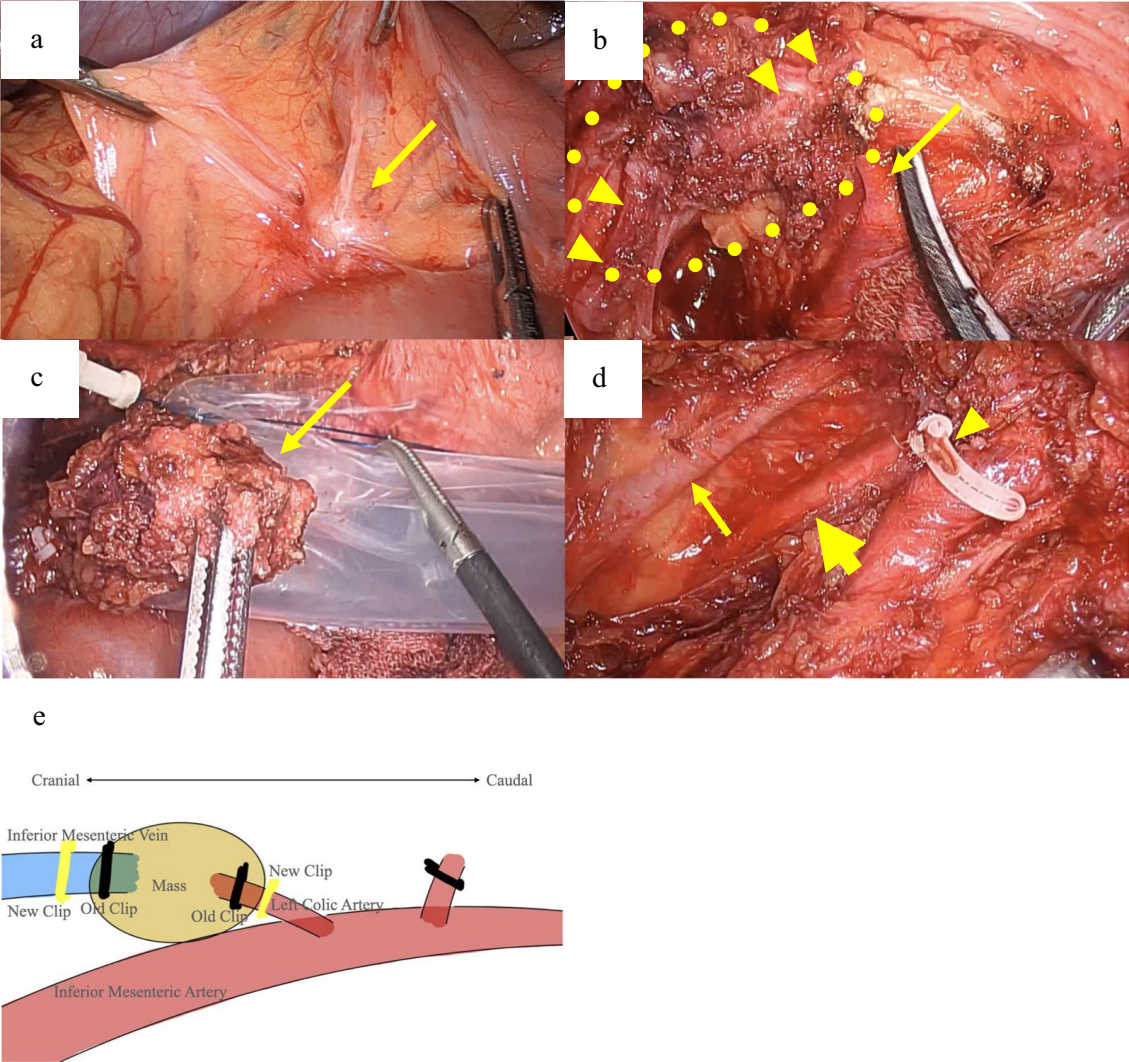
Intraoperative findings. **a** Intraoperative identification of the mass (arrow) in the mesocolon lateral to the duodenum. **b** Visualization of the two polymer clips (arrowheads) attached to the mass (encircled with a dotted line), with the inferior mesenteric artery and root of the left colic artery (arrow) revealed. **c** Resection of the mass (arrow). **d** No residual lesion in the area, with the left colic artery (arrowhead) clipped at its origin from the inferior mesenteric artery, and preservation of the gonadal vessel (thin arrow) and ureter (thick arrow). **e** Illustration depicting the vessel anatomy, clips, and the mass. New clips (drawn in yellow) are applied to the left colic artery and inferior mesenteric vein to excise the mass along with the old clips (drawn in black)

**Fig. 5:**
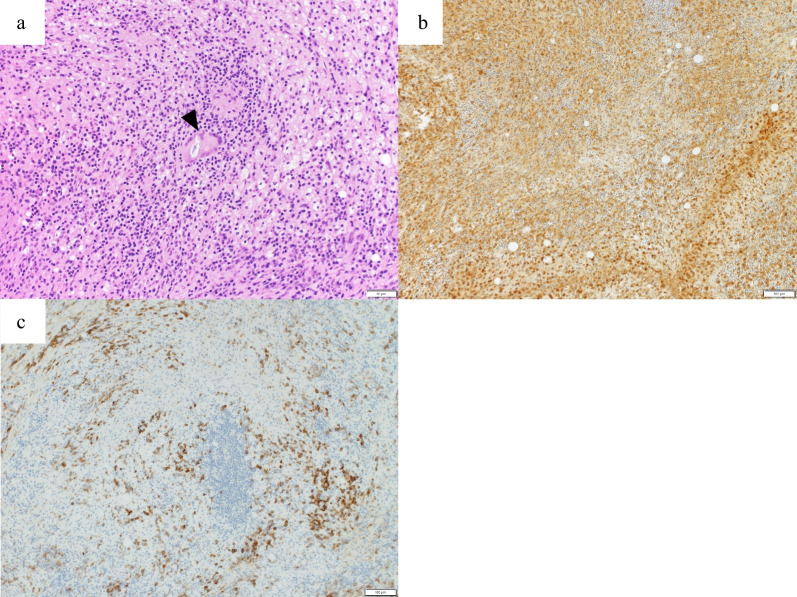
Histopathological examinations of the resected specimen. **a** Hematoxylin and eosin staining revealing scattered multinucleated giant cells (arrowhead) (*200). **b** CD68 staining indicating numerous macrophages (*100). **c** CD138 staining showing abundant plasma cells (*100). The specimen exhibits granulation tissue characteristics

A follow-up CT, 2 years postoperatively, revealed a 10 mm mass adjacent to the IMA (Fig. 2a). Although the CEA was 6.1 ng/mL with no elevation, a further positron emission tomography (PET)/CT was recommended considering the possibility of lymph node recurrence. It showed increased fluorodeoxyglucose (FDG) uptake, with a maximum standardized uptake value (SUVmax) of 10.6 (Fig. 2b). No anastomotic recurrence was detected on colonoscopy; however, a repeat CT scan 3 months later showed that the mass had grown to 15 mm (Fig. 3a, b), suspicious of recurrence. Considering the initial cancer’s pStage I status and slight decrease in CEA to 5.1 ng/mL during this period, a differential diagnosis of benign lesions such as a desmoid tumor was considered. Core needle biopsy was deemed impossible because of the location of the mass, leading to laparoscopic surgical resection for a definitive diagnosis. The decision for surgical resection was made with anticipation of potential infiltration into the duodenum, necessitating extensive resection, possibly including duodenectomy. Intraoperatively, a solid mass was identified within the mesocolon on the lateral side of the duodenum (Fig. 4a). The mass adhered to the surrounding tissue, making it difficult to determine its border. An extensive dissection was performed on the anterior surface of the abdominal aorta and around the IMA to expose the LCA root. The two polymer clips used in the initial surgery for LCA and IMV ligation were attached to the mass (Fig. 4b). Subsequently, new clips were applied to the root of the LCA and further centrally to the IMV, and the mass was excised together with the old clips (Fig. 4c–e). The surgery lasted for 161 min with minimal blood loss. The final pathology report confirmed that a foreign body granuloma had formed in reaction to the polymer clips and was composed of granulation tissue with numerous macrophages, lymphocytes, plasma cells, and multinucleated giant cells (Fig. 5a–c). No evidence of malignancy was found, and a desmoid tumor was excluded with negative results of β-catenin. Neither mycobacteria nor fungi were identified with Ziehl–Neelsen or periodic acid–Schiff staining. The patient had a favorable postoperative course, with no evidence of recurrence at the latest follow-up after 2 months. The patient was informed of the necessity of using clips or sutures for vessel ligation, despite the potential risk of inducing granulomatous reactions.

## Discussion

We identified two noteworthy clinical issues: nonabsorbable polymer clips can induce a granulomatous reaction, and clinical differentiation from lymph node recurrence can be challenging, especially in patients with a history of cancer.

First, polymer clips can cause foreign body granulomas several months after surgery, as any foreign body within the body can trigger a granulomatous reaction [10]. An intravital experiment on mice skin proposed the following sequence of events: macrophages bind to the surface of the foreign body and differentiate into giant cells; once these giant cells fuse together, they produce vascular endothelial growth factor-A. This in turn recruits fibroblasts to encapsulate the foreign body in the collagen matrix [11]. Our review of case reports on PubMed using keywords “foreign body granuloma” and “colon cancer or colorectal cancer” revealed that silk sutures were primarily responsible for most cases of foreign body granuloma following colorectal cancer surgery [1–5, 7, 12–14]. Table 1 shows the recent English case reports of foreign body granuloma after colorectal cancer surgery with the present case indicated as No. 11 for comparison. Other reports of foreign body granuloma after general surgery mentioned those elicited by polypropylene sutures, surgical sponges, and metal clips [12, 15, 16]. One letter has described a case similar to ours, in which a polymer Hem-O-Lok® clip caused a foreign body granuloma [17]. This demonstrates the significance of categorizing such cases as “clip granulomas” for future recognition.

**Table 1:** Recent English case reports of foreign body granuloma after colorectal cancer surgery

No.	Year	Author	Age/sex	Primary cancer location	Stage	Duration from last surgery (month)	Mass location	FDG-PET, SUVmax	Preoperative diagnosis	Definitive diagnostic method	Foreign body
1	2009	Kim [1]	67/M	R	I	30	Pelvic wall	9.1	Recurrence	Excisional biopsy	Suture
2	2009	Kim [1]	37/M	T + LM	IV	3	Liver resection site	9.6	Liver metastasis	Partial resection of the liver	NA
3	2015	Nihon-Yanagi [12]	66/M	S + LM	IV	19	Liver resection site	Not taken	Recurrence	Laparotomy	Metal clips
4	2016	Matsuura [2]	71/M	S + LM	IV	4	Below the diaphragm	5.48	Peritoneal dissemination	Partial hepatectomy, partial resection of the diaphragm	Silk suture
5	2016	Pantiora [13]	48/F	NA + LM	IV	12	Liver resection site	8	Recurrence	Laparotomy	NA
6	2016	Martínez-Martínez [3]	66/M	S	ND	3	Retroperitoneum	5.04	Tumor implant	Surgical resection	Suture
7	2019	Asano [14]	85/M	S	IIIa	10	Abdominal wall	14.6	Port site recurrence	Surgical resection	NA
8	2021	Huang [4]	60/M	A	II	24	Mesocolon near the anastomosis	Not taken	Local recurrence	Laparotomy	Silk suture
9	2021	Takano [5]	63/M	R	I	29	Mesocolon near the anastomosis	Negative	Recurrence	Laparotomy	Silk suture
10	2024	Mitsuyoshi [7]	66/F	A	IIIA	NA	Port site	Positive	NA	Laparoscopic resection	Sutures
11	2024	Our case	47/F	S	I	12	Vascular stump of the mesocolon	10.9	Lymph node recurrence, desmoid tumor	Laparoscopic resection	Polymer clip

*M* male, *F* female, *A* ascending colon, *R* rectum, *S* sigmoid colon, *T* transverse colon, *LM* liver metastasis, *NA* not available, *FDG* fluorodeoxyglucose, *PET* positron emission tomography, *SUVmax* maximum standardized uptake value

In the present case, in contrast to the clips applied on the LCA and IMV, the clip on the S1 did not cause noticeable granulation. The factors distinguishing these outcomes remain unknown; our hypothesis is that the clip on the LCA, being slightly apart from the bifurcation, potentially had more contact with the surrounding tissues and thus induced a more robust inflammatory response.

Second, a mass resembling a lymph node recurrence may be a granuloma formed in reaction to vessel clips. Both malignant tumors and inflammatory granulomas exhibit increased FDG accumulation. A meta-analysis of focal colorectal incidental FDG uptake revealed substantial overlap in the average standardized uptake values among malignant, premalignant, and benign lesions [18]. Our literature review on intra-abdominal foreign body granulomas suggested a median SUVmax value of 8.55 (5.04–14.6) (Table 1). Although the cutoff value for an intra-abdominal mass is unknown, a previous review recommended using SUVmax > 11.4 to distinguish adenocarcinoma from other etiologies of focal colonic uptake, with 80% sensitivity and 82% specificity [19]. This implied a potentially higher SUVmax for malignant lesions than for benign lesions. Additionally, certain features of PET/CT scans aid in differentiation. One such feature is ring-shaped FDG uptake in foreign body granulomas, which reflects the thick wall of the granuloma and the encapsulated foreign body [20]. Another indication is that cancer cells show increased uptake in the later phase, whereas benign tumors do not [21]. In our case, the clips were not completely encapsulated by the granuloma but rather attached to it, resulting in an atypical homogenous appearance. Furthermore, a dual-time PET/CT scan was not performed, making it impossible to distinguish benign granulomas from lymph node recurrence.

Another differential diagnosis of a lymph node recurrence mimic is desmoid tumor, which is a benign fibroblastic neoplasm that grows slowly. Up to 30% of the cases are associated with previous injuries or surgery [22], which can make it challenging to distinguish them from cancer recurrence, particularly in patients with a history of surgical resection. We have identified several English case reports of intra-abdominal desmoid tumors after colorectal cancer surgery, which were detected from 6 to 36 (median, 18) months post-surgery [23–29]. However, PET/CT was performed only in three cases, all showing the SUVmax values below 5 [23, 27, 28]. Upon the detection of an enlarging mass on postoperative cancer follow-up CT, it is imperative to raise concerns regarding recurrence. However, alternative possibilities must be discussed, especially in low-risk situations, such as pStage I colon cancer (4.4% recurrence rate after curative resection) [30]. In our case, the suspicion of a desmoid tumor was unique (Table 1) and was possibly attributable to the early postoperative period and low recurrence risk.

To establish a definitive diagnosis, excisional biopsy is recommended for deep lesions to minimize the risk of cancer dissemination during incisional biopsy. Indeed, almost all previous intra-abdominal cases were conclusively diagnosed via surgical resection (Table 1) [1–9, 12–14]. Only a few exceptions involving core needle biopsies were found in cases where a benign tumor was strongly suspected based on the typical imaging findings of a foreign body granuloma [16]. In cases where sacrificing surrounding organs is required for complete mass resection, considering intraoperative frozen section diagnosis from partial resection can help minimize the extent of surgery. In conclusion, if percutaneous biopsy is impractical due to the location of the mass and the possibility of cancer recurrence cannot be entirely ruled out, opting for excisional biopsy is the best possible choice. This proactive approach, as employed in our case, aims to remove the mass before it infiltrates surrounding organs, thereby minimizing the extent of necessary resection. This strategy not only facilitates a more accurate diagnosis, but also contributes to a more conservative treatment plan for the patient.

## Conclusions

This case report highlights the importance of considering foreign body granulomas in patients who have undergone surgery with polymer clips. It also emphasizes that lymph node recurrence mimics might be foreign body granulomas. Although surgical resection and pathological examination are essential for a definitive diagnosis, this awareness can guide clinicians to achieve accurate assessments and deliver appropriate explanation and treatment to their patients. Further research is warranted to explore the predisposing factors that make patients more susceptible to foreign body granulomas and determine the specific surgical materials that provoke robust granulomatous reactions so as to plan strategic preparations for alternative devices.

## Data Availability

Data presented in this case report are available from the corresponding author upon reasonable request.

## Abbreviations

CT: Computed tomography
IMA: Inferior mesenteric artery
SRA: Superior rectal artery
LCA: Left colic artery
S1: First sigmoid artery
IMV: Inferior mesenteric vein
CEA: Carcinoembryonic antigen
PET: Positron emission tomography
FDG: Fluorodeoxyglucose
SUVmax: Maximum standardized uptake value
